# Multimodal processing in face-to-face interactions: A bridging link between psycholinguistics and sensory neuroscience

**DOI:** 10.3389/fnhum.2023.1108354

**Published:** 2023-02-02

**Authors:** Stefania Benetti, Ambra Ferrari, Francesco Pavani

**Affiliations:** ^1^Centre for Mind/Brain Sciences, University of Trento, Trento, Italy; ^2^Interuniversity Research Centre “Cognition, Language, and Deafness”, CIRCLeS, Catania, Italy; ^3^Max Planck Institute for Psycholinguistics, Donders Institute for Brain, Cognition, and Behaviour, Radboud University, Nijmegen, Netherlands

**Keywords:** multimodal communication, face-to-face interactions, social actions, lateral cortical processing pathway, psycholinguistics, sensory neuroscience

## Abstract

In face-to-face communication, humans are faced with multiple layers of discontinuous multimodal signals, such as head, face, hand gestures, speech and non-speech sounds, which need to be interpreted as coherent and unified communicative actions. This implies a fundamental computational challenge: optimally binding only signals belonging to the same communicative action while segregating signals that are not connected by the communicative content. How do we achieve such an extraordinary feat, reliably, and efficiently? To address this question, we need to further move the study of human communication beyond speech-centred perspectives and promote a multimodal approach combined with interdisciplinary cooperation. Accordingly, we seek to reconcile two explanatory frameworks recently proposed in psycholinguistics and sensory neuroscience into a neurocognitive model of multimodal face-to-face communication. First, we introduce a psycholinguistic framework that characterises face-to-face communication at three parallel processing levels: multiplex signals, multimodal gestalts and multilevel predictions. Second, we consider the recent proposal of a lateral neural visual pathway specifically dedicated to the dynamic aspects of social perception and reconceive it from a multimodal perspective (“lateral processing pathway”). Third, we reconcile the two frameworks into a neurocognitive model that proposes how multiplex signals, multimodal gestalts, and multilevel predictions may be implemented along the lateral processing pathway. Finally, we advocate a multimodal and multidisciplinary research approach, combining state-of-the-art imaging techniques, computational modelling and artificial intelligence for future empirical testing of our model.

## Introduction

In face-to-face communication, we encounter multiple layers of discontinuous multimodal signals: head, face, mouth movements, hand gestures, speech and non-speech sounds. This implies a fundamental computational challenge: optimally binding only signals belonging to the same communicative action while segregating unrelated signals (Noppeney, 2021). Within this challenge, the temporal misalignment of fast-changing signals across different sensory channels raises a central binding problem (Chen and Vroomen, 2013). Finally, each conversational partner is taxed by fast turn-taking dynamics (Levinson, 2016). Despite these critical constraints, we process multimodal communicative signals faster than speech alone (Holler et al., 2018; Drijvers and Holler, 2022). Crucially, we use non-verbal communicative signals to facilitate semantic understanding (Özyürek, 2014) and pragmatic inference (Holler, 2022). How do we achieve such an extraordinary feat?

To address this question, we need to move beyond the prominent speech-centred research perspective on the neurocognitive mechanisms of human communication. Building on previous calls for the need to study language in its multimodal manifestation and ecological context (Levinson and Holler, 2014; Vigliocco et al., 2014; Hasson et al., 2018; Perniss, 2018), the view we put forward here seeks to reconcile two explanatory frameworks recently proposed in psycholinguistics and sensory neuroscience. Specifically, we first highlight that verbal and non-verbal communicative signals are integrated to represent socially relevant acts (Levinson and Holler, 2014) through domain-general mechanisms of multimodal integration and prediction (Holler and Levinson, 2019). Accordingly, we then reconceive the neuroscientific evidence of a third visual pathway, specialised for dynamic aspects of social perception (Pitcher and Ungerleider, 2021), from a multimodal perspective. Finally, we propose that the resulting brain network implements the sensory processing gateway necessary toward successful multimodal processing and interpretation of face-to-face communicative signals.

## Multimodal processing in face-to-face interactions: A possible computational framework

**FIGURE 1:**
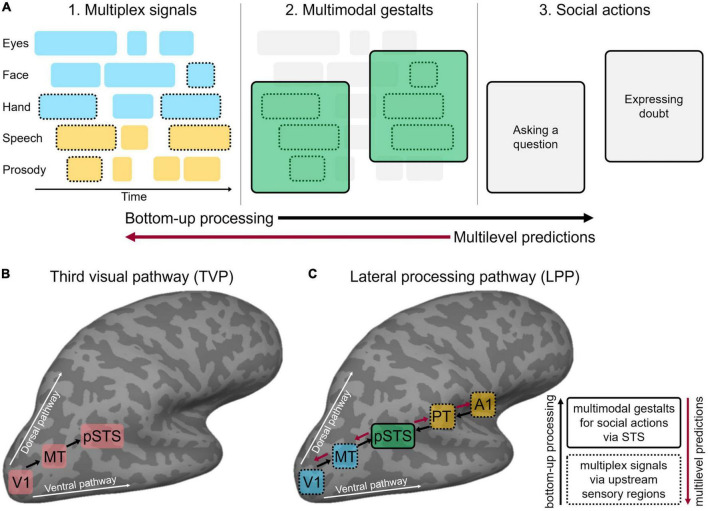
**(A)** Face-to-face communication comprises multiple layers of discontinuous multimodal signals emitted by different articulators (eyes, face, etc.) over time (visual in cyan, auditory in yellow). To enable effective communication, interlocutors must bind only signals belonging to the same communicative action while segregating tangential, unrelated signals that do not share the communicative content. Temporal statistical regularities allow chunking coherent communicative inputs into multiplex signals (dashed contours) at a perceptual, pre-semantic level (A1). Further, statistical regularities between multiplex signals and communicative meanings generate multimodal gestalts (solid contours) that bear semantic and pragmatic value (A2) and thus signal a specific communicative intention in conversation, i.e., social action (A3). Finally, social action recognition may trigger top-down multilevel predictions across hierarchically organised linguistic and perceptual levels. Following a parallel processing framework, concurrent bottom-up sensory processing (black arrow) and multilevel predictions (red arrow) iteratively refine each other. **(B)** Schematic representation of the third visual cortical pathway (TVP) specialised for the dynamic aspects of social perception, as proposed by Pitcher and Ungerleider (2021). The pathway originates in the primary visual cortex (V1) and dissociates from both the ventral and dorsal pathways by projecting into the posterior portion of the superior temporal sulcus (pSTS) via motion-selective areas (MT). **(C)** Schematic representation of the multimodal lateral processing pathway (LPP) implementing the sensory processing gateway toward successful face-to-face communication, as proposed in the present perspective. The LPP originates in early visual (V1) and auditory (A1) areas and dissociates from ventral and dorsal pathways by projecting to the mid-posterior portion of STS via animacy and motion-selective areas (here, MT and PT only are represented for clarity of visualisation). Black arrows indicate bottom-up processing along the LPP hierarchy from upstream regions (dashed contours) responsible for multiplex signals (panel A1) to portions of the pSTS (solid contours) that contribute toward the implementation of multimodal gestalts (panel A2). Red arrows indicate top-down multilevel predictions via pSTS to upstream visual and auditory areas (in cyan and yellow, respectively). Panel **(A)** is based on Holler and Levinson, 2019.

Holler and Levinson (2019) recently outlined the key computational principles that support fast and efficient multimodal processing in face-to-face communication, with the ultimate goal of interpreting communicative social actions (Figure 1A). First, domain-general mechanisms of multimodal integration (Stein, 2012; Noppeney, 2021) are hypothesised to be co-opted for detecting communicative signals. For example, faster processing of multimodal relative to unimodal communicative inputs mirrors multimodal facilitation outside the domain of communication in humans (Murray et al., 2001; Senkowski, 2005; Diederich et al., 2009) and animals (Gingras et al., 2009). Holler and Levinson (2019) proposed that multimodal interactions resting on statistical regularities among sensory inputs allow chunking the stream of concurrent dynamic inputs into *multiplex signals* at a perceptual, pre-semantic level. Further, the statistical regularities between multiplex signals and communicative meanings generate *multimodal gestalts* that bear semantic and pragmatic value, thus signalling a specific social action. For example, eyebrow frowns often accompany a raising voice pitch to signal the intention to ask a question (Nota et al., 2021). Mechanisms of Gestalt perception (Wagemans et al., 2012), social affordance (Gallagher, 2020), and relevance (Sperber and Wilson, 1995) may jointly contribute to the recognition of multimodal communicative gestalts (Trujillo and Holler, 2023). Finally, the recognition of a specific social action may trigger top-down *multilevel predictions* about how the message will unfold in time. For example, frowning and pointing at an object typically anticipates a question about that object, triggering top-down hierarchical predictions at multiple sensory levels (e.g., vocal sounds, bodily movements) and linguistic levels (e.g., words, sentential units). Multiplex signals, multimodal gestalts, and multilevel predictions are thought to interact in a continuous, dialectic process, leading to incremental unification while the message unfolds (Hagoort, 2005, 2019). Specifically, this supports a parallel processing framework whereby the beginning of the message simultaneously activates multiple potential interpretations (i.e., multimodal gestalts). As the message unfolds, concurrent bottom-up sensory processing and multilevel predictions iteratively refine each other toward a final gestalt solution (Trujillo and Holler, 2023). Such a parallel account accommodates evidence that processing of communicative social actions starts early (Redcay and Carlson, 2015), perhaps in parallel to semantic comprehension (Tomasello et al., 2022).

Supporting this framework, there is substantial psycholinguistic evidence for systematic associations between facial-bodily signals and social actions (Holler and Levinson, 2019; Nota et al., 2021). Moreover, the early emergence of these perceptual associations in infants (Cameron-Faulkner et al., 2015), as well as parallels in non-human primates (Rossano and Liebal, 2014), suggest they might be deeply rooted in the human onto- and phylogenesis.

## Multimodal processing in face-to-face interactions: A possible neural framework

Accumulating evidence (Pitcher et al., 2014; Walbrin and Koldewyn, 2019; Landsiedel et al., 2022) suggests that dynamic visual aspects of social perception (e.g., face, hand and body movements across the visual field) cannot be easily accommodated within the classic dual-stream model for visual perception (Ungerleider and Mishkin, 1982). Accordingly, resting on both anatomical and functional evidence in humans and non-human primates, Pitcher and Ungerleider (2021) proposed the existence of a third visual processing pathway (Figure 1B) that projects on the lateral cortical surface from the early visual cortex into the mid-posterior superior temporal sulcus (pSTS) *via* motion-selective occipito-temporal areas (V5/hMT). Consistent evidence shows that pSTS preferentially responds to multiple types of dynamic social bodily inputs including eye, mouth, hands, and body movements (Allison et al., 2000; Hein and Knight, 2008; Deen et al., 2020). Importantly, both anterior hMT (Desimone and Ungerleider, 1986; Huk et al., 2002) and pSTS (Bruce et al., 1981; Pitcher et al., 2020; Finzi et al., 2021) respond to dynamic signals across both visual hemifields in human and non-human primates, in opposition to the contralateral field bias that characterises the ventral pathway (Finzi et al., 2021). Together, these functional properties are thought to support social interaction, which is an inherently dynamic process requiring the integration of sensory information across the entire visual field (Pitcher and Ungerleider, 2021).

Relevantly, Pitcher and Ungerleider (2021) note that the *“proximity (to pSTS, a.n.) of brain areas computing multisensory information relevant to social interactions further dissociates the third pathway from the established role of the ventral and dorsal pathways.”* We further elaborate on this by reconceiving the third visual pathway as a fundamental part of a larger multimodal neural system that implements fast analysis of multisensory communicative signals during face-to-face interactions. This pathway projects from early visual and auditory regions along the lateral brain surface and into the pSTS (lateral processing pathway; LPP). From this perspective, regions in the mid-posterior and lateral superior temporal gyrus, which are sensitive to auditory motion, animacy, sounds of moving bodies and dynamic aspects of human vocalisation (i.e., prosodic intonation), become candidate nodes of the auditory bank of LPP.

Analogously to the third visual pathway, evidence supporting the existence of a third lateral auditory cortical pathway, independent of dorsal/ventral pathways (Rauschecker, 1998; Rauschecker and Tian, 2000) and projecting *via* motion-sensitive regions into the posterior STS, comes from both tracer studies in macaques and *in vivo* white matter tractography in humans (see Table 1, connectivity profiles). These mid-posterior lateral areas showing anatomical connectivity with the pSTS also show motion-sensitive and voice-sensitive responses, suggesting functional selectivity for dynamic biologically-relevant information along this lateral auditory pathway (see Table 1, functional properties). Relevantly, functional interactions and direct anatomical connections have also been observed between auditory and visual motion-sensitive regions (see Table 1), suggesting a structural scaffolding for early convergence of multimodal information (Benetti and Collignon, 2022) within temporo-occipital regions of the LPP that might share the same computational goal: fast and reliable analysis of multimodal information relevant to social interactions.

**TABLE 1:** Functional properties and structural connectivity profile of mid-posterior and lateral auditory areas in the superior temporal gyrus as described in (a) non-human and (b) human primates.

Auditory area	Functional/Connectivity profile	References
**(a) In non-human primates**		
Mid-posterior parabelt	Auditory motion processing	Poirier et al., 2017
Mid-lateral parabelt	Processing of conspecific vocalization	Petkov et al., 2008; Perrodin et al., 2011
Mid-posterior parabelt	Connection to the mid-posterior STS	Galaburda and Pandya, 1983; Hackett et al., 1998; de la Mothe et al., 2006; Hackett et al., 2007; Smiley et al., 2007
Motion-sensitive areas	Monosynaptic connection to visual MT	Ungerleider and Desimone, 1986; Boussaoud et al., 1990; Palmer and Rosa, 2006
**(b) In human primates**		
Bilateral hPT	Preferential processing of moving sounds	Krumbholz et al., 2005; Battal et al., 2019
Right lateral hPT	Responses to ipsilateral auditory field	Krumbholz et al., 2005
Bilateral anterior hPT	Encoding of living and human-action sounds categories	Giordano et al., 2013
Right anterior hPT and area adjacent to TVA	Responses to socially meaningful prosody	Belyk and Brown, 2014; Sammler et al., 2015; Hellbernd and Sammler, 2018
Bilateral lateral hPT	White matter connections to mid- and posterior upper bank of STS	Beer et al., 2013
Bilateral mid-lateral STG	White matter connections to mid-upper bank of STS	Beer et al., 2013
Bil. motion-selective portions of hPT	White matter connections to motion-selective hMT	Gurtubay-Antolin et al., 2021

STS, superior temporal sulcus; MT, middle temporal visual area; hPT, human planum temporale; TVA, temporal voice area; STG, superior temporal gyrus; Bil., Bilateral.

## Toward a neurocognitive model of face-to-face communication

In the following section, we attempt to reconcile the psycholinguistic (Holler and Levinson, 2019) and sensory neuroscience (Pitcher and Ungerleider, 2021) frameworks, reviewed so far, toward a coherent neurocognitive model of multimodal face-to-face communication. Accordingly, we propose how key computational principles underlying the perception of multimodal social actions (multiplex signals, multimodal gestalts, and multilevel predictions) might be implemented along the LPP (Figure 1C).

### Detecting multimodal co-occurrences: Multiplex signals *via* upstream sensory regions

Traditionally, it was thought that multimodal integration takes place in higher-order polysensory areas such as parietal or prefrontal cortices, after unimodal processing in early sensory regions (Ungerleider and Mishkin, 1982; Rauschecker and Tian, 2000); however, accumulating evidence over the past two decades shows clear cross-modal interactions between early sensory areas (Foxe and Schroeder, 2005; Ghazanfar and Schroeder, 2006; Kayser and Logothetis, 2007; Driver and Noesselt, 2008). In fact, several studies with humans (Foxe et al., 2000, 2002; Schürmann et al., 2006; Martuzzi et al., 2007; Besle et al., 2008; Lewis and Noppeney, 2010) and primates (Schroeder et al., 2001; Fu et al., 2003; Kayser et al., 2005, 2008; Lakatos et al., 2007) have proved driving or modulatory effects of cross-modal stimuli at the bottom of the sensory processing hierarchy. Beyond identifying multimodal interactions, such evidence also revealed their ubiquity across the (sub)cortical hierarchy and called for the need to further characterise the computational principles, neural properties and behavioural relevance of these interactions. One possibility is that they differ at different processing stages (i.e., *multistage integration*) along the (sub)cortical hierarchy (Calvert and Thesen, 2004; Noppeney et al., 2018; Noppeney, 2021).

Since visual bodily signals typically precede speech during natural face-to-face interactions (Nota et al., 2021), they may modulate the sound-induced activity in the auditory cortex by resetting the phase of ongoing oscillations (Biau et al., 2015; Mégevand et al., 2020; Pouw et al., 2021). In support of a temporally-sensitive mechanism, neurophysiological (Kayser et al., 2010; Atilgan et al., 2018), and fMRI studies (Lewis and Noppeney, 2010; Werner and Noppeney, 2011) have shown that audiovisual interactions in early auditory cortex and hPT depended on audiovisual temporal coincidence or coherence over time. Sensitivity to temporal co-occurrences is crucial to multiplex signals, which rest on temporal statistical regularities across sensory channels at a perceptual, pre-semantic level (Holler and Levinson, 2019). Therefore, it seems plausible that upstream sensory regions (e.g., visual and auditory cortices) interact in a temporally-sensitive fashion at corresponding processing stages (i.e., *via* multistage integration) to implement multiplex signals [see also Bizley et al. (2016)]. Specifically, it may be that primary visual and auditory cortices concur to support the automatic, salience-driven detection of multimodal co-occurrences, while secondary visual and auditory cortices along the LPP (hMT/EBA and hPT/TVA) concur to represent dynamic aspects of audiovisual bodily signals, mirroring results outside the realm of face-to-face communication (Lewis and Noppeney, 2010).

### Recognizing communicative meanings: Multimodal gestalts *via* pSTS

As reviewed above, upstream visual and auditory sensory regions are structurally and functionally interconnected with pSTS. Crucially, this region represents a site of multimodal integration of social and non-social sensory information, as shown in neuroimaging and neurophysiological studies with humans (Beauchamp, 2005; Beauchamp et al., 2008; Werner and Noppeney, 2010a,b; Hirsch et al., 2018; Noah et al., 2020) and non-human primates (Ghazanfar et al., 2008; Froesel et al., 2021). While these studies employed non-linguistic but meaningful world categories such as animals, manipulable objects, and human actions, pSTS is also involved in the processing of communicative and meaningful audiovisual stimuli such as lip-speech (MacSweeney et al., 2000; Wright, 2003; Macaluso et al., 2004; van Atteveldt et al., 2004; Stevenson and James, 2009; Price, 2012; Venezia et al., 2017) and gesture-speech (Holle et al., 2008, 2010; Hubbard et al., 2009; Willems et al., 2016). Consistently, multimodal integration in pSTS may allow the creation of meaningful neural representations (Beauchamp et al., 2004; Noppeney et al., 2018), including those bearing semantic and pragmatic values for social communication (i.e., multimodal gestalts; Holler and Levinson, 2019). In particular, we propose that pSTS might concur toward such (multimodal) neural representations based on Bayesian Causal Inference principles (Körding et al., 2007; Shams and Beierholm, 2010; Noppeney, 2021), mirroring effects found along the dorsal audiovisual pathways for spatial localisation (Rohe and Noppeney, 2015, 2016; Aller and Noppeney, 2019; Ferrari and Noppeney, 2021).

Intriguingly, pSTS is positioned at the intersection of three brain systems respectively responsible for social perception, action observation, and theory of mind (Yang et al., 2015). As noticed by Pitcher and Ungerleider (2021), perceptual analysis of goal-directed actions in the pSTS likely influences activity in parietal and frontal systems that are responsible for action and intention recognition. As such, after receiving converging inputs from upstream sensory regions of the LPP, pSTS may represent the sensory processing gateway that feeds to higher-order networks for social action recognition during face-to-face communication. As a result, multiplex signals may be processed at the semantic and pragmatic levels, enabling the recognition of multimodal gestalts (Holler and Levinson, 2019).

### Predicting how the conversation unfolds: Multilevel predictions along the cortical hierarchy

Increasing evidence shows that humans, among other species, build on their past experiences to construct predictive models of themselves and their sensory environment (de Lange et al., 2018). Accordingly, the brain can be conceived as a “prediction machine” (Clark, 2013) that attempts to match bottom-up sensory inputs with top-down expectations. Following hierarchical predictive coding (Rao and Ballard, 1999; Friston, 2005, 2010), any mismatch between expectation and actual input is signalled as a prediction error that propagates up the processing hierarchy to higher-level areas; vice versa, expected inputs are “explained away,” resulting in “expectation suppression” (Summerfield et al., 2008; Alink et al., 2010; Richter et al., 2018; Walsh et al., 2020). Importantly, expectation suppression reflects the neural tuning properties along a given processing hierarchy. For example, predictions about visual object and face identity are associated with expectation suppression respectively in object-selective regions (Meyer and Olson, 2011; Kaposvari et al., 2018; Richter et al., 2018; Ferrari et al., 2022; He et al., 2022) and face-selective regions (Summerfield et al., 2008; Amado et al., 2016; Schwiedrzik and Freiwald, 2017) along the ventral visual stream [for corresponding effects in the auditory domain, see e.g., Jaramillo and Zador (2011), Todorovic et al. (2011), Barascud et al. (2016), Heilbron and Chait (2018)].

Similarly, multilevel predictions during face-to-face interactions (Holler and Levinson, 2019) may be implemented *via* mechanisms of hierarchical predictive processing in neural pathways that are responsible for coding the relevant sensory information (e.g., vocal sounds, bodily movements) and linguistic information (e.g., words, sentential units, social actions). Increasing evidence shows signatures of hierarchical predictive processing during language comprehension in left-lateralized fronto-temporal regions of the language network (Blank and Davis, 2016; Sohoglu and Davis, 2016; Willems et al., 2016; Schmitt et al., 2021; Heilbron et al., 2022). Accordingly, predictive processing mechanisms may implement multimodal sensory predictions relevant to face-to-face interactions along the cortical hierarchy of the LPP. Initial evidence shows that hMT and pSTS activity is reduced in response to expected than unexpected visual actions (Koster-Hale and Saxe, 2013), such as human movements violating biomechanical predictions (Costantini et al., 2005; Saygin et al., 2012). Further, pSTS activity is reduced in response to actions that fit rather than violate the spatiotemporal structure of the environment (Koster-Hale and Saxe, 2013), such as shifting head and gaze toward rather than away an abrupt warning signal (Pelphrey et al., 2003). Interestingly, there is evidence of a functional dissociation between hMT and pSTS, with only the latter being sensitive to violations of action intentions (Pelphrey et al., 2004). Such dissociation is suggestive of a hierarchy of computations from sensory processing of dynamic inputs in hMT (at the level of multiplex signals) to semantic and pragmatic analysis in pSTS (at the level of multimodal gestalts), which may then be reflected in the respective expectation suppression profiles. Yet, it remains an open question whether and how multimodal (e.g., audiovisual) predictions arising from face-to-face interactions generate neural signatures of hierarchical predictive processing along the entire LPP, down to upstream sensory regions [for complementary evidence, see Lee and Noppeney (2014)]. Further, it is unknown whether and how higher-order expectations from language, action recognition and theory of mind networks may feed-back to pSTS (Yang et al., 2015) and thus travel down the LPP.

## Discussion and conclusion

The current proposal leaves many aspects of the model un- or under-specified, including issues of hemispheric lateralization (Pitcher and Ungerleider, 2021) and the exact relationship between LPP and brain networks responsible for language (Hickok and Poeppel, 2000, 2007; Friederici, 2012; Hagoort, 2019), action recognition (Lingnau and Downing, 2015; Wurm and Caramazza, 2022), and theory of mind (Frith and Frith, 2006; Mar, 2011; Schaafsma et al., 2015). Future research must provide direct empirical evidence to support our framework, as well as refine and enrich it at the algorithmic and neural levels. To start, neuroimaging and neurostimulation techniques may characterise the functional and representational properties of the LPP as proposed here, as well as its degree of lateralization and interconnection with other brain networks (Thiebaut de Schotten and Forkel, 2022). Further, it will be crucial to combine these techniques with methodological approaches that enable human motion-tracking and near-to-optimal preservation of naturalistic, ecological contexts of face-to-face social interactions, such as virtual reality (Peeters, 2019). Complementarily, hyperscanning (Redcay and Schilbach, 2019; Hamilton, 2021) and multibrain stimulation techniques (Novembre and Iannetti, 2021) will be necessary to probe the functional relevance of the LPP during multimodal face-to-face processing across interacting brains. In parallel, the use of computational models (e.g., Bayesian Causal Inference) and neuroscientific-inspired artificial intelligence (i.e., convolutional or deep neural networks) could formalise the empirical evidence and test its role (e.g., necessity, sufficiency) for human behaviour (Hassabis et al., 2017) during face-to-face interactions. Last, but not least, it will be crucial to further embrace an interdisciplinary perspective in which psycholinguistics and neuroscientific frameworks would be reciprocally validated.

We conclude that the time is mature to accept the challenge we, among others before, advocated in this perspective and move beyond the speech-centred perspective dominating research on the neurocognitive mechanisms of human communication and language. We offer an original perspective bridging two recent propositions in psycholinguistics (Holler and Levinson, 2019) and sensory neuroscience (Pitcher and Ungerleider, 2021) into a neurocognitive model of multimodal face-to-face communication. Testing this framework represents a novel and promising endeavour for future research.

## Author contributions

SB and AF contributed equally to the original conception of the perspective and wrote the first draft of the manuscript. FP contributed to further developing the preliminary conception. All authors contributed to manuscript revision, read, and approved the submitted version.
